# Supplementary material for: Laminin signals initiate the reciprocal loop that informs breast-specific gene expression and homeostasis by activating NO, p53 and microRNAs
Source: eLife. 2018 Mar 21;7:e26148. doi: 10.7554/eLife.26148 (PMC5862529; doi:10.7554/eLife.26148)
Supplement: Transparent reporting form [file elife-26148-transrepform.pdf]

1st Floor
24 Hills Road
Cambridge CB2 1JP, UK

P 01223 855340
W elifesciences.org
T @elife

eLife's transparent reporting form

We encourage authors to provide detailed information *within their submission* to facilitate the interpretation and replication of experiments. If you have any questions, please contact us: editorial@elifesciences.org.

Sample-size estimation

- You should state whether an appropriate sample size was computed when the study was being designed
- You should state the statistical method of sample size computation and any required assumptions
- If no explicit power analysis was used, you should describe how you decided what sample (replicate) size (number) to use

Please outline where this information can be found within the submission (e.g., page numbers or figure legends), or explain why this information doesn't apply to your submission:

For each analysis, appropriate sample size and power analyses were computed with online calculators (Chow et al., 2008; Musonda et al., 2006) using the formula described (Kadam et al., 2010). The sample size of each analysis is included in the corresponding figure legend as follows:

Fig. 1c (p49); Fig. 2b, 2d (p49); Fig. 3a-3d (p50); Fig. 4a- 4e (p51); Fig. 5a, 5b (p52); Fig. 6c; Fig. 7a-7h (p54); Fig. 8a-8e (p55); Fig. 9b-9d (p56)

Fig. S1 (p58); Fig. S2c (p58); Fig. S3b-S3e (p58-59); Fig. S4a-S4f (p59-60); Fig. S5a-S5d (p60); Fig. S6a, S6b (p61); Fig. S7a-S7c (p61-62);Fig. S8a-c (p62); Fig. S9a-c (p62)

References:

Chow S, Shao J, Wang H. Sample Size Calculations in Clinical Research. Clinical Research 2nd Ed Chapman & Hall/CRC Biostatistics Series. 2008.

Musonda P, Farrington CP, Whitaker HJ. Sample sizes for self-controlled case series studies. Statistics in Medicine 2006;25:2618-31.

Kadam, P., Bhalerao, S. Sample size calculation. Int. J. Ayurveda Res. 2010; 1(1): 55–57

Replicates

- You should report how often each experiment was performed
- You should include a definition of biological versus technical replication

eLife Sciences Publications, Ltd is a limited liability non-profit non-stock corporation incorporated in the State of Delaware, USA, with company number 5030732, and is registered in the UK with company number FC030576 and branch number BR015634 at the address 1st Floor, 24 Hills Road, Cambridge CB2 1JP | August 2014

- The data obtained should be provided and sufficient information should be provided to indicate the number of independent biological and/or technical replicates
- If you encountered any outliers, you should describe how these were handled
- Criteria for exclusion/inclusion of data should be clearly stated
- High-throughput sequence data should be uploaded before submission, with a private link for reviewers provided (these are available from both GEO and ArrayExpress)

Please outline where this information can be found within the submission (e.g., page numbers or figure legends), or explain why this information doesn't apply to your submission:

For each analysis, replicate experiments (n=3) were performed, and representative data are shown. In addition, each experiment contained at least 3 biological replicates, and measurement was performed at least 3 times to obtain 3 technical replicates.

Outliers were defined as those with the deviations which were twice the standard deviation of the rest of the population. These samples were removed from the data analyses.

Microarray analysis was deposited in GEO (GSE50444, https://www.ncbi.nlm.nih.gov/geo/query/acc.cgi?acc=GSE50444).

This information is found in each figure legend, Materials and Methods as follows:

Figure Legends
Fig. 1a, c, Fig. 2a-d, Fig. 3a-d, Fig. 4a-d, Fig. 5a-h, Fig. 6a-c, Fig. 7a-h, Fig. 8a-e, Fig. 9a-c. Fig. 10a-d. Fig. S1b-e, Fig. S2a-c, Fig. S3a-d, Fig. S4 a-f, Fig. S5a, Fig. S6a, b, Fig. S7a-c, Fig. S8a-c, Fig. S9a-c

Statistical reporting

- Statistical analysis methods should be described and justified
- Raw data should be presented in figures whenever informative to do so (typically when N per group is less than 10)
- For each experiment, you should identify the statistical tests used, exact values of N, definitions of center, methods of multiple test correction, and dispersion and precision measures (e.g., mean, median, SD, SEM, confidence intervals; and, for the major substantive results, a measure of effect size (e.g., Pearson's r, Cohen's d)
- Report exact p-values wherever possible alongside the summary statistics and 95% confidence intervals. These should be reported for all key questions and not only when the p-value is less than 0.05.

Please outline where this information can be found within the submission (e.g., page numbers or figure legends), or explain why this information doesn't apply to your submission:

Statistical analysis method were described in Materials Methods under Statistics (p28).

For each analysis, replicate experiments (n>3) were performed, and representative data are shown. Data measurements were performed at least 3 times to obtain at least n=10 sample numbers. Therefore, we did not include the raw data of these analyses.

All the statistical analyses were performed using Graph Pad Prism Version 5 software and an unpaired 2-tailed Student's t-test for parametric tests and Spearman correlation analysis for non-parametric tests. P-values of 0.05 or less were considered significant. Average results of multiple experiments (n>3) are presented as the arithmetic mean±SEM. This information is included in the Materials and Methods.

We did not indicate the exact p-values of most of analyses because of space constraint. However, we used the universal annotations as "*" for p<0.05, "**" for p<0.01 and "***" for p<0.001 within the graphs.

However, for some of critical information (e.g., miRNA level), we did include the exact p-values in Table 2 and 3.

(For large datasets, or papers with a very large number of statistical tests, you may upload a single table file with tests, Ns, etc., with reference to page numbers in the manuscript.)

Additional data files ("source data")

- We encourage you to upload relevant additional data files, such as numerical data that are represented as a graph in a figure, or as a summary table
- Where provided, these should be in the most useful format, and they can be uploaded as "Source data" files linked to a main figure or table
- Include model definition files including the full list of parameters used
- Include code used for data analysis (e.g., R, MatLab)
- Avoid stating that data files are "available upon request"

Please indicate the figures or tables for which source data files have been provided:

None
